# Supplementary material for: Distinct neuropeptide-receptor modules regulate a sex-specific behavioral response to a pheromone
Source: Commun Biol. 2021 Aug 31;4:1018. doi: 10.1038/s42003-021-02547-7 (PMC8408276; doi:10.1038/s42003-021-02547-7)
Supplement: Supplementary file 2 — Supplementary Information [file 42003_2021_2547_MOESM2_ESM.pdf]

Distinct Neuropeptide-Receptor Modules Regulate a Sex-Specific Behavioral Response to a Pheromone

Douglas K. Reilly, Emily J. McGlame, Elke Vandewyer, Annalise M. Robidoux, Caroline S. Muirhead, Haylea T. Northcott, William Joyce, Mark J. Alkema, Robert J. Gegear, Isabel Beets, Jagan Srinivasan*

Supplementary Figure and Table Legends

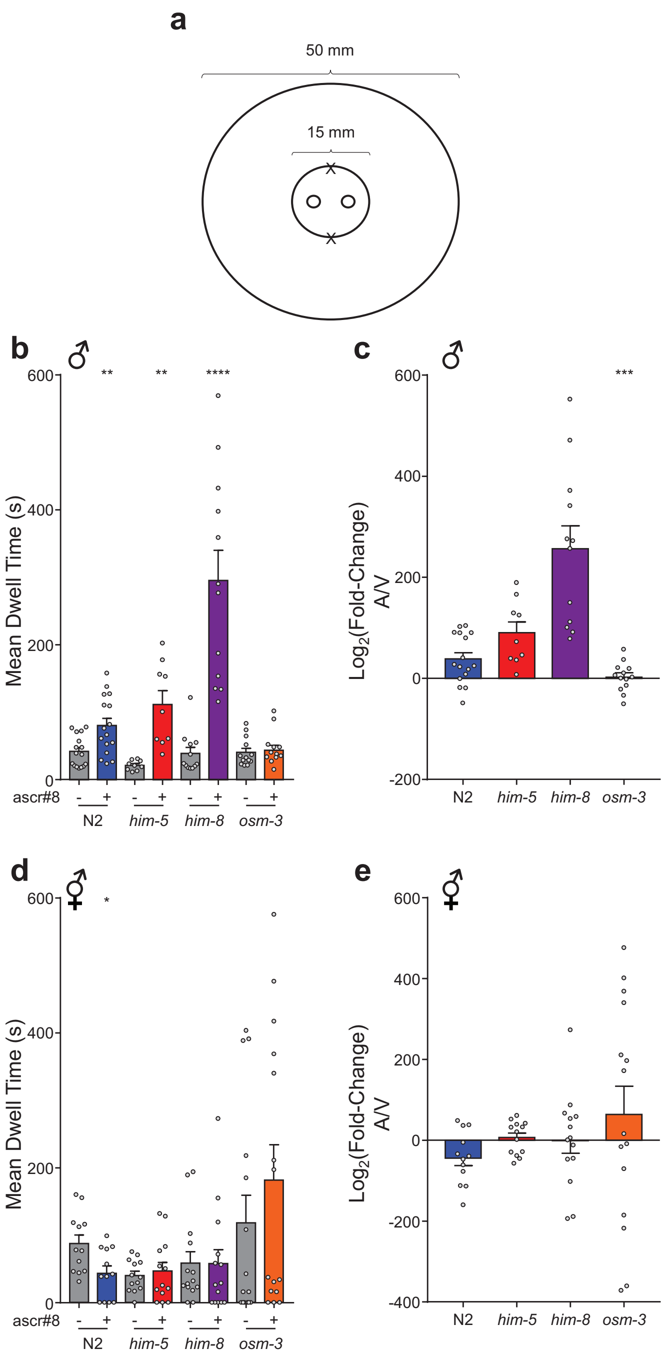

Supplementary Figure 1. Canonical Spot Retention Assay. (a) The Spot Retention Assay (SRA), as described previously [14,33]. **(b)** Male *C. elegans* are attracted to ascr#8 in all wild-type strains (N2, *him-5*, *him-8*), but not chemosensory mutants (*osm-3;him-5*). **(c)** Transformed attraction data, logbase2(fold-change) [38] , of the raw data shown in **(b)**. **(d)** Hermaphroditic responses to ascr#8 across strains. **(e)** Transformed logbase2(fold-change) data of hermaphrodite SRA data. Grey denotes vehicle control ("-"), while colors denote ascr#8 ("+") dwell time (N2, blue; *him-5*, red; *him-8*, purple; *osm-3;him-5*, orange). Error bars denote SEM. $n \geq 10$. * $p < 0.05$, ** $p < 0.01$, *** $p < 0.001$, **** $p < 0.0001$. **(b,d)** Paired *t*-test of vehicle vs. ascr#8. **(c,e)** One-Way ANOVA, followed by Dunnett's Correction. ♂ denotes male data, ⚥ denotes hermaphrodite data.

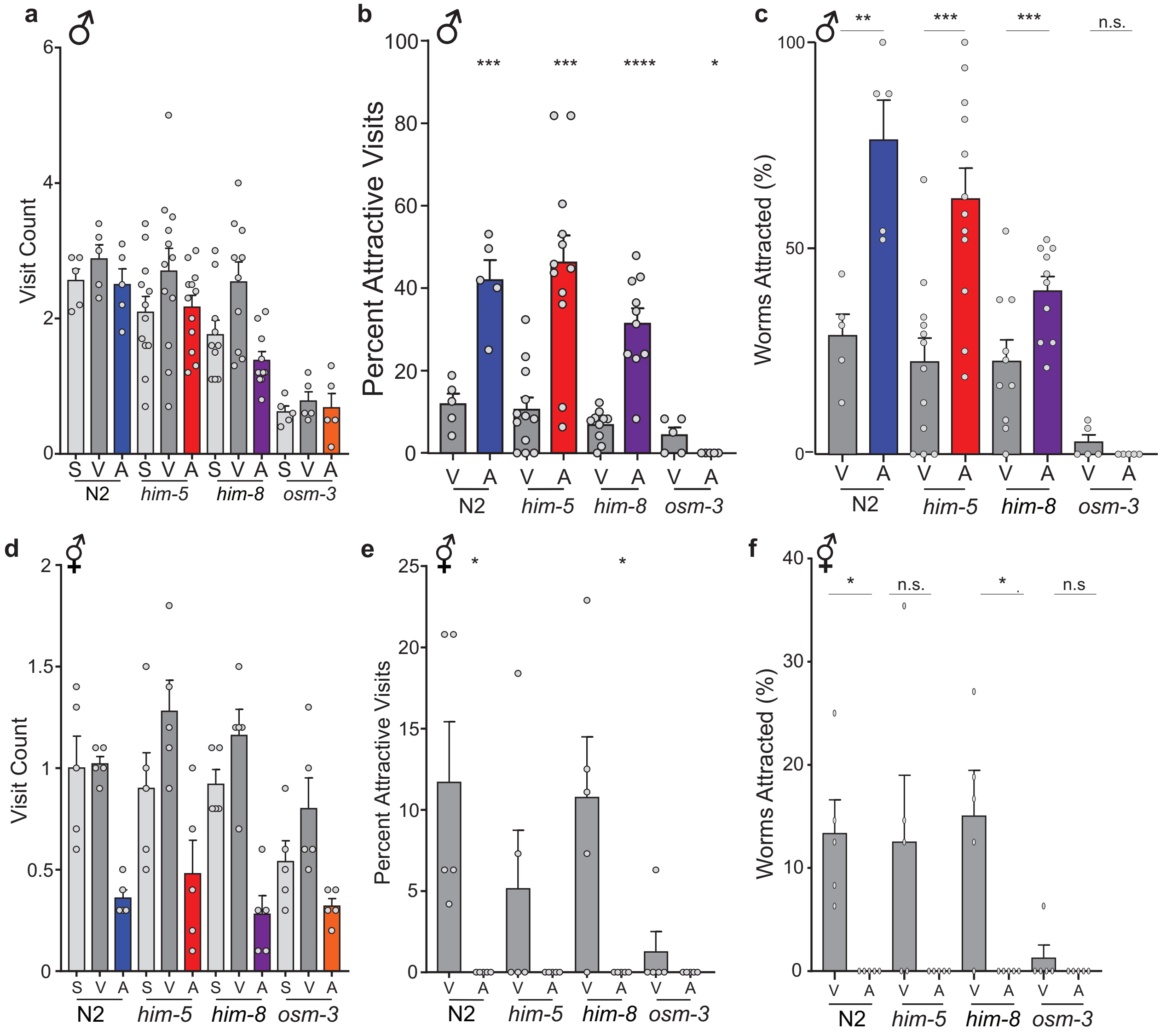

Supplementary Figure 2. Single Worm Attraction Assay of control strain visit count and percent attractiveness. (a) Male visit count of control strains. **(b)** Percent of attractive visits per worm for males of control strains. **(c)** Percentage of male worms that exhibited any attractive visits. **(d)** Hermaphrodite visit count of control strains. **(e)** Percent of attractive visits per worm of control strains. **(f)** Percentage of hermaphrodite worms that exhibited any attractive visits. Light grey denotes spatial controls ("S") (when applicable), dark grey denotes vehicle controls ("V"), colors denote ascr#8 values ("A") (N2, blue; *him-5*, red; *him-8*, purple; *osm-3;him-5*, orange). Error bars denote SEM. $n \geq 5$. * $p < 0.05$, ** $p < 0.01$, *** $p < 0.001$, **** $p < 0.0001$. **(a,c)** RM-ANOVA, followed by Bonferroni's Correction. **(b, d, e, f)** Paired *t*-test of V vs. A. ♂ denotes male data, ⚥ denotes hermaphrodite data.

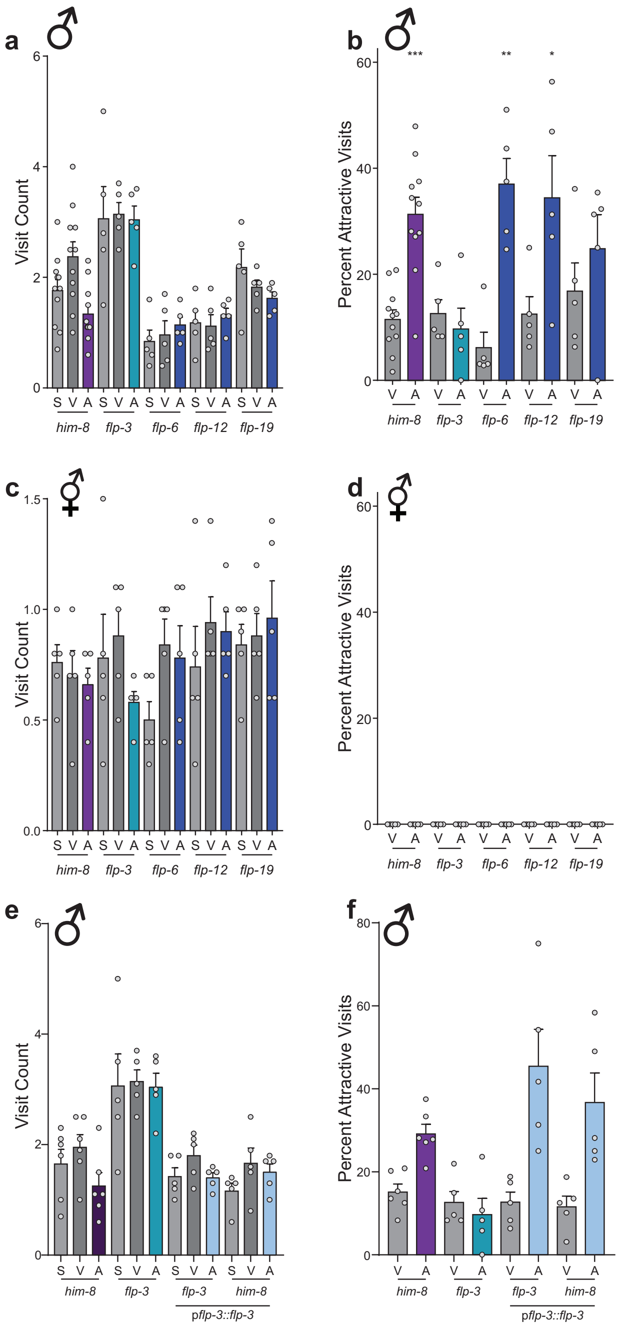

Supplementary Figure 3. Neuropeptide null mutant screen SWAA Supplementary information. (a) Visit counts for males defective in neuropeptide genes. **(b)** Percent of attractive visits per worm of males defective in neuropeptide genes. **(c)** Hermaphrodite visit counts. **(d)** Percent of hermaphrodite attractive visits per worm. **(e)** Visit counts for males of *flp-3* rescue and overexpression transgenics. **(f)** Percent of attractive visits per male worm of *flp-3* rescue and overexpression transgenics. Light grey denotes spatial controls ("S") (when applicable), dark grey denotes vehicle controls ("V"), colors denote ascr#8 values ("A") (*him-8*, purple; *flp-3*, teal; *flp* mutants, blue; transgenic rescues, light blue). Error bars denote SEM**.** $n \geq 5$. * $p < 0.05$, ** $p < 0.01$, *** $p < 0.001$, **** $p < 0.0001$. **(a,c,e)** RM-ANOVA, followed by Bonferroni's Correction. **(d,e,f)** Paired *t*-test of V vs. A. ♂ denotes male data, ⚥ denotes hermaphrodite data.

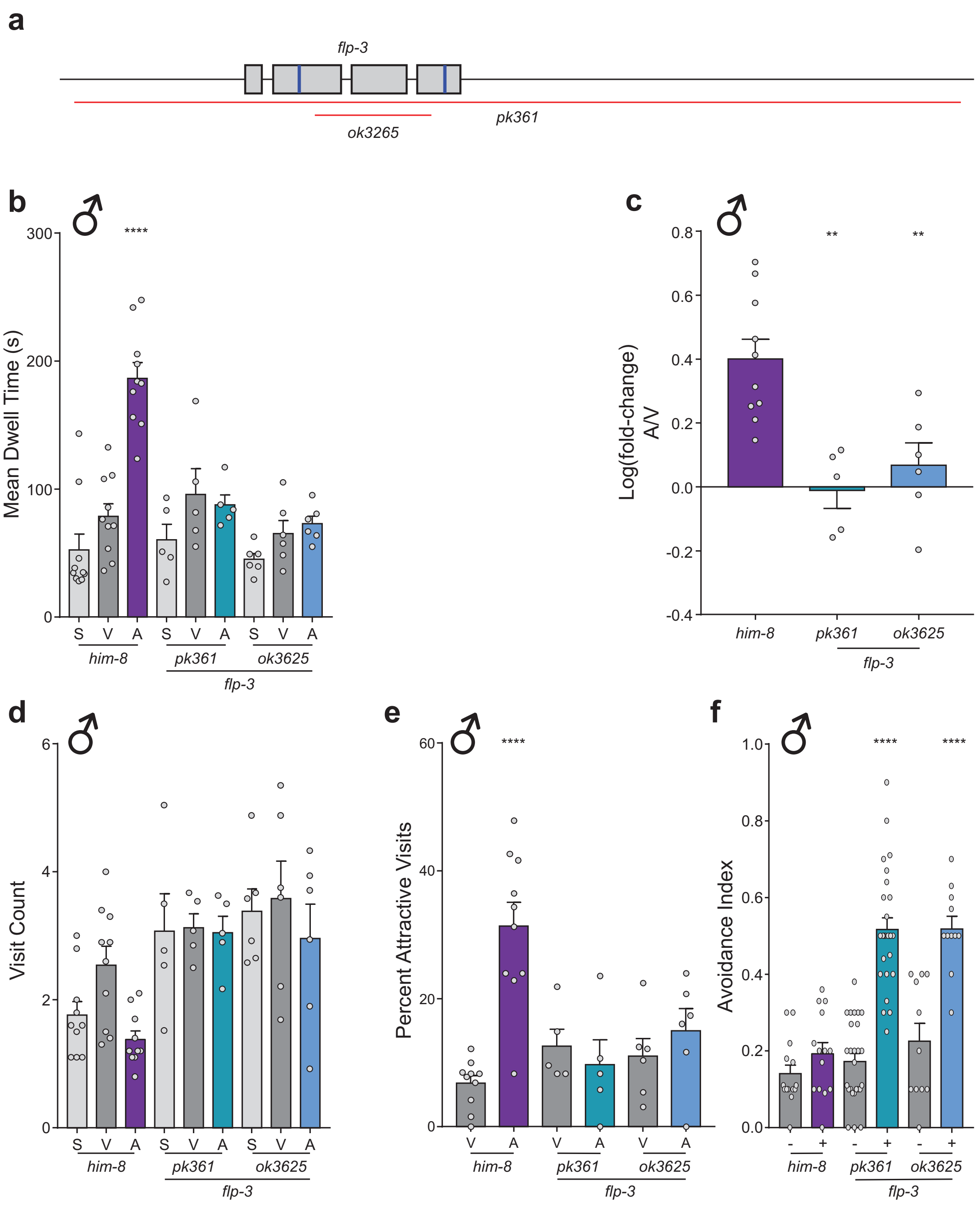

Supplementary Figure 4. The behavioral phenotypes in *flp-3 lof* males are consistent across different alleles. (a) Schematic of *flp-3* deletion alleles *pk361* and *ok3625*, which result in a full gene deletion and in-frame partial gene deletion, respectively (red lines). **(b)** Raw dwell time, **(c)** log(fold-change) values, **(d)** visit count, and **(e)** percent of attractive visits of both *flp-3* alleles, *pk361* and *ok3625*. **(f)** Avoidance indexes of both *flp-3* alleles. Light grey denotes spatial controls ("S") (when applicable), dark grey denotes vehicle controls ("V"), colors denote ascr#8 values ("A") (*him-8*, purple; *flp-3(pk361)*, teal; *flp-3(ok3625)*, light blue). Error bars denote SEM. $n \geq 5$. * $p < 0.05$, ** $p < 0.01$, *** $p < 0.001$, **** $p < 0.0001$. **(a,d)** RM-ANOVA followed by Bonferroni's Correction. **(c)** One-Way ANOVA followed by Dunnett's Correction. **(e,f)** Paired *t*-test of **(e)** V vs. A or **(f)** – vs. +. ♂ denotes male data.

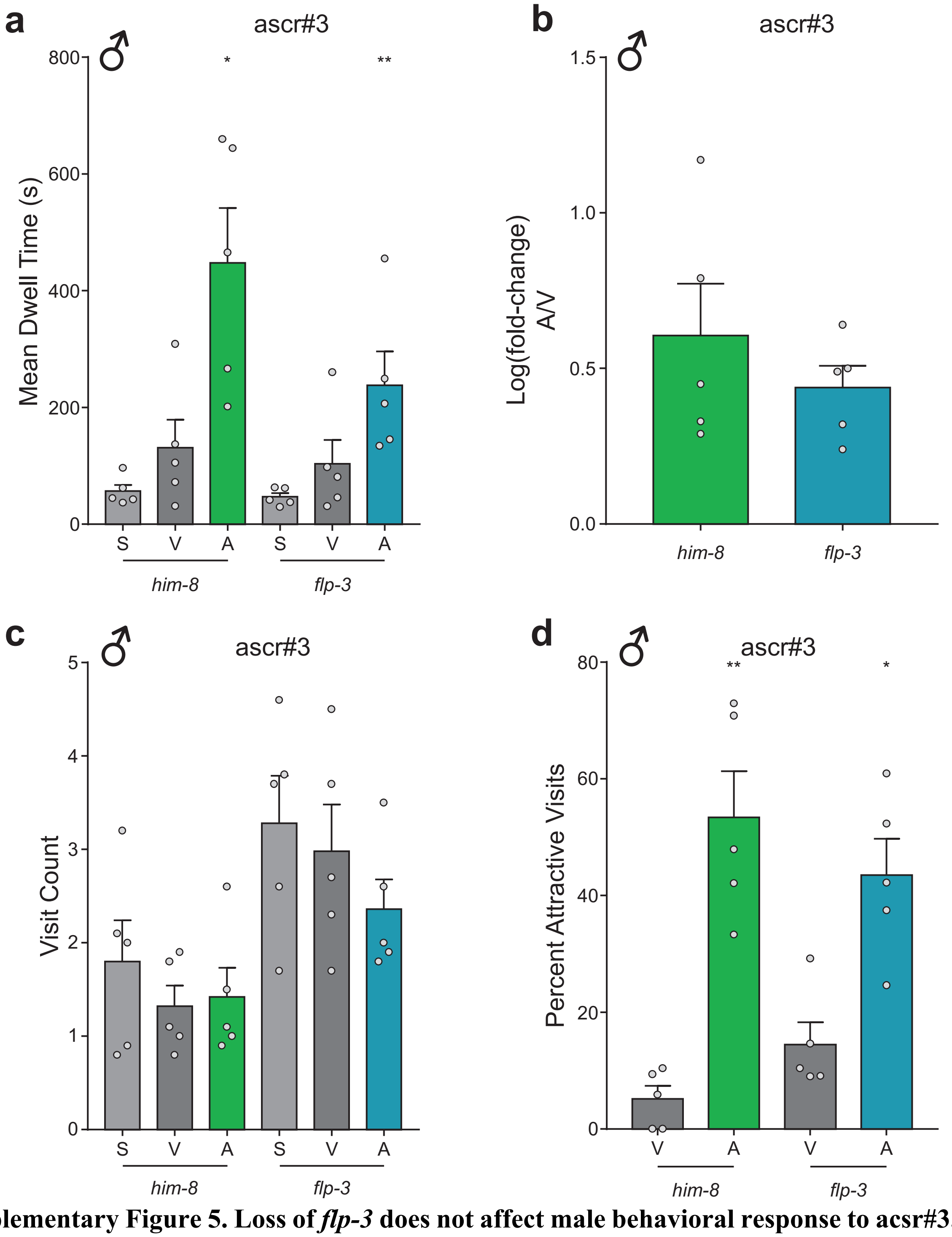

Supplementary Figure 5. Loss of *flp-3* does not affect male behavioral response to acsr#3. (a) Raw dwell time, **(b)** log(fold-change) values, **(c)** visit count, and **(d)** percent of attractive visits of *flp-3(pk361)* to ascr#3. Light grey denotes spatial controls ("S") (when applicable), dark grey denotes vehicle controls ("V"), colors denote ascr#8 values ("A") (*him-8*, green; *flp-3*, teal). Error bars denote SEM**.** $n = 5$. * $p < 0.05$, ** $p < 0.01$. **(a,c)** RM-ANOVA followed by Bonferroni's Correction. **(b,d)** Paired *t*-test of **(b)** log(fold-change) or **(d)** V vs. A. ♂ denotes male data.

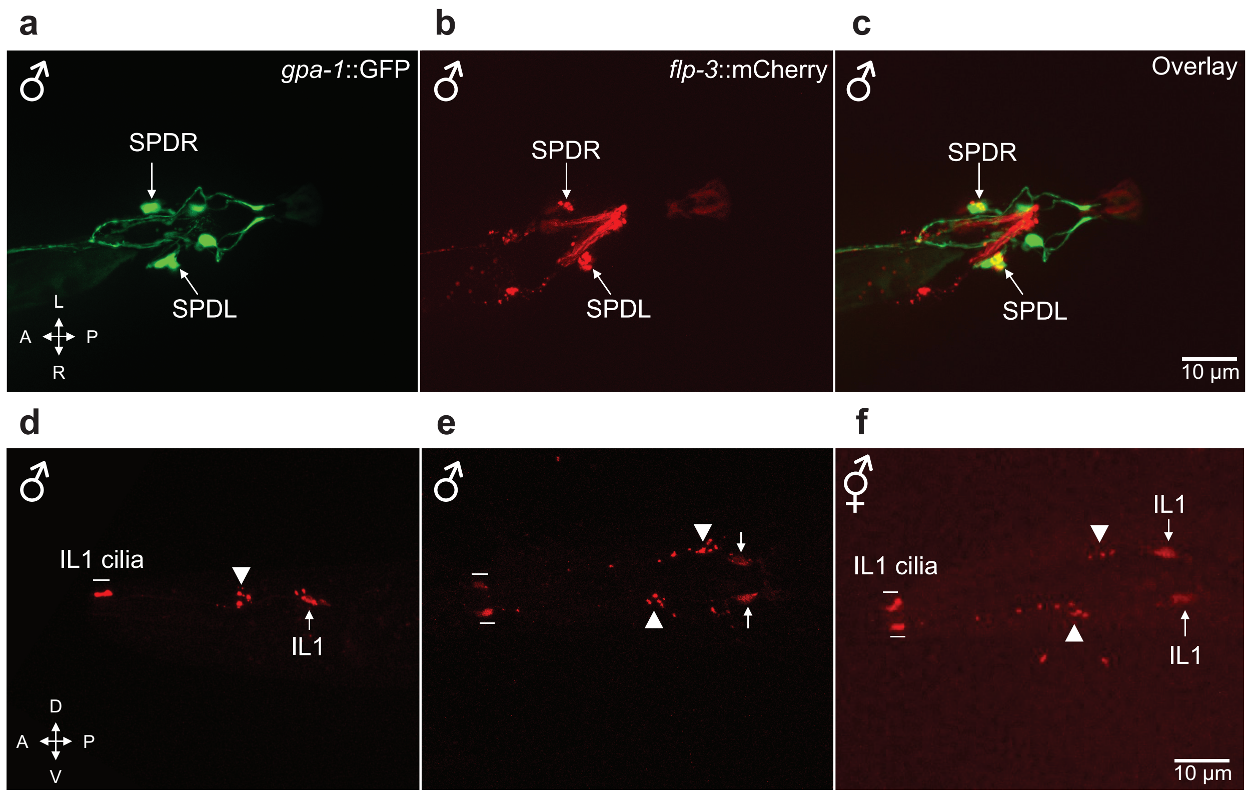

Supplementary Figure 6. Expression pattern analysis of p*flp-3*::*flp-3*::mCherry. (a-c) ~90x magnification of a mail tail expressing **(a)** *gpa-1*::GFP and **(b)** p*flp-3*::*flp-3*::mCherry. **(c)** The two reporters co-localize in the SPD neuronal soma (arrows). **(d-f)** IL1 expression of p*flp-3*::*flp-3*::mCherry. mCherry is faintly observed in the IL1 soma (arrows). The fluorescent protein is also observed in the dendritic cilia of the IL1 neurons (bars), as well as in punctate vesicles along the dendrites (arrowheads). ♂ denotes male data, ⚥ denotes hermaphrodite data.

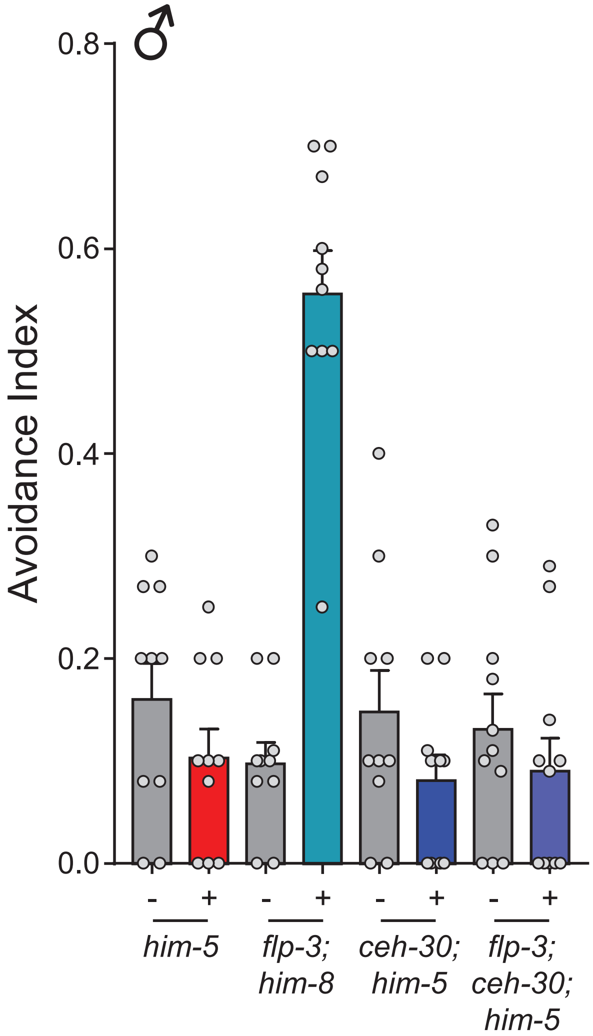

Supplementary Figure 7. The male-specific cephalic sensory CEM neurons are necessary detecting ascr#8 in males. Avoidance index values of *him-5* and *flp-3;him-8* control males, as well as *ceh-30;him-5* and *ceh-30;flp-3;him-5* males. Grey bars depict avoidance to vehicle control, colors denote mutant avoidance to ascr#8. *him-5* males (red) do not avoid ascr#8, while *flp-3* males (teal) do. The loss of the CEM neurons in the *ceh-30* mutant (blue) does not result in attraction. Loss of both chemosensory pathways and *flp-3* neuromodulation (*flp-3;ceh-30*, blue) does not result in avoidance to ascr#8. Grey denotes vehicle controls ("-"), colors denote ascr#8 values ("+") (*him-8*, red; *flp-3*, teal; *ceh-30*, dark blue; *flp-3;ceh-30*, blue). Error bars denote SEM**.** $n \geq 10$. . Paired *t*-test of vehicle vs. ascr#8 ("-" vs. "+"). ♂ denotes male data.

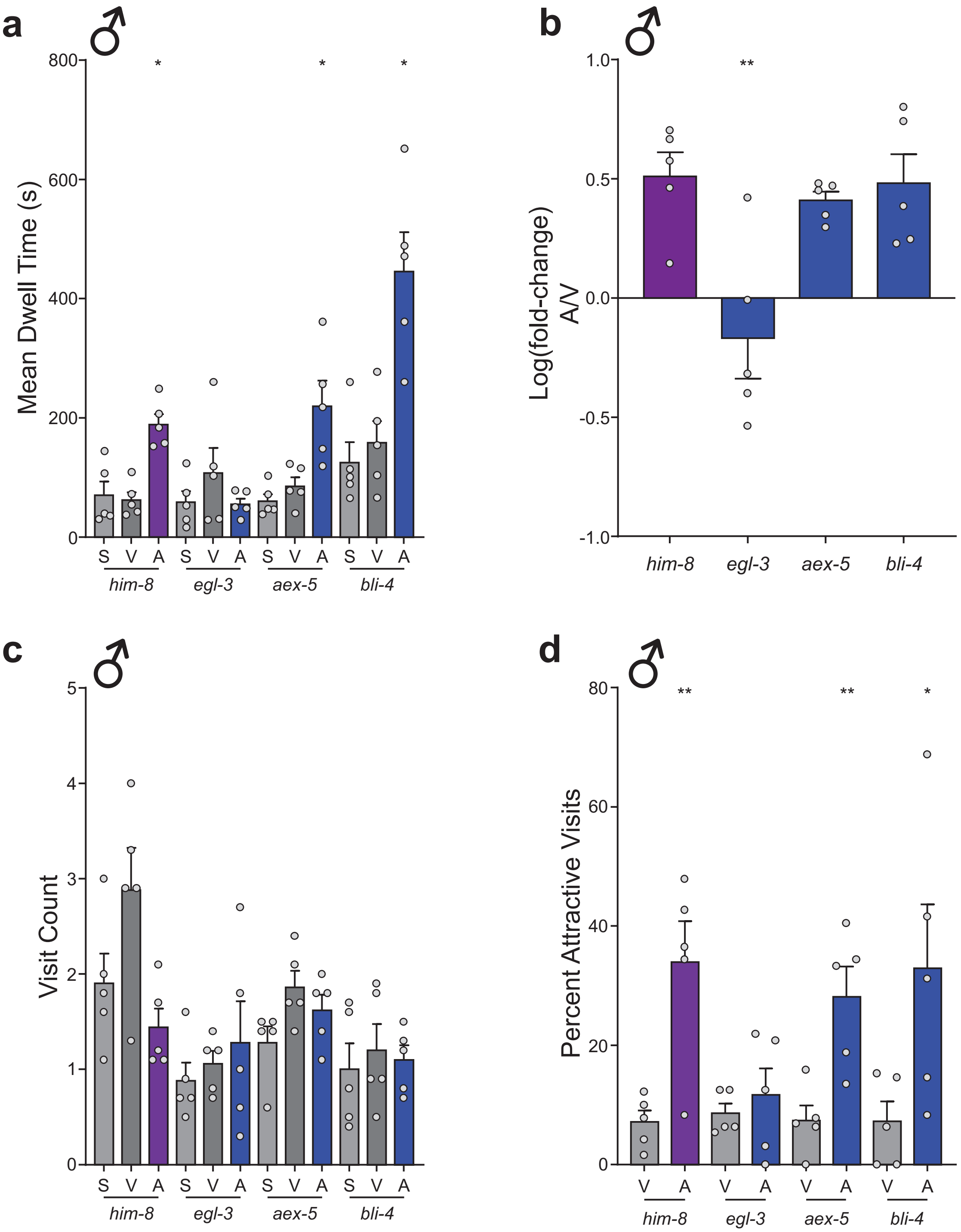

Supplementary Figure 8. EGL-3 is the proprotein cleavage enzyme responsible for FLP-3 maturation. **(a)** Raw dwell time, **(b)** log(fold-change) values, **(c)** visit count, and **(d)** percent of attractive visits per worm of propeptide convertase enzymes involved in neuropeptide maturation: *egl-3*, *aex-5*, and *bli-4*. Light grey denotes spatial controls ("S") (when applicable), dark grey denotes vehicle controls ("V"), colors denote ascr#8 values ("A") (*him-8*, purple; enzyme mutants, blue). Error bars denote SEM**.** $n = 5$. * $p < 0.05$, ** $p < 0.01$. **(a,c)** RM-ANOVA, followed by Bonferroni's Correction. **(b)** One-Way ANOVA, followed by Dunnett's Correction. **(d)** Paired *t*-test of V vs. A. ♂ denotes male data.

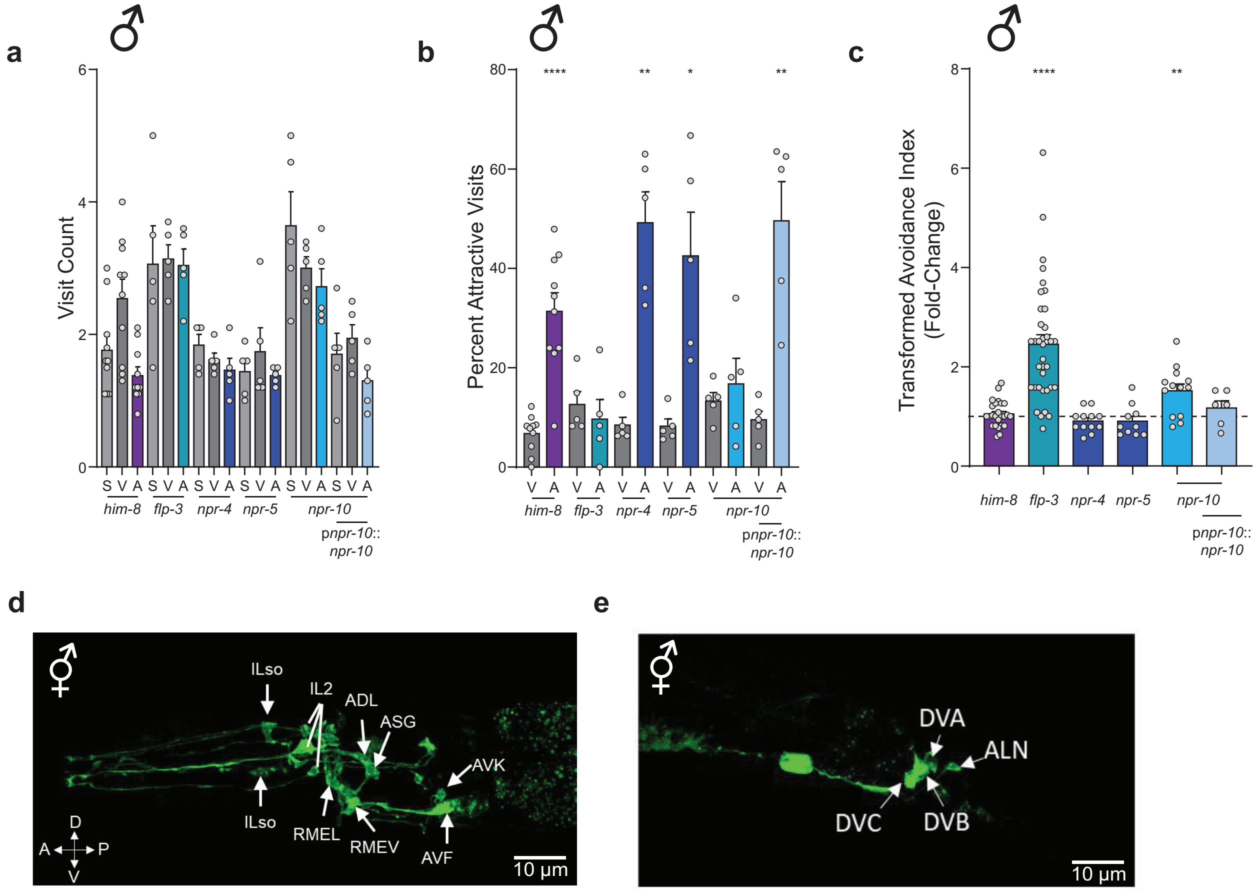

Supplementary Figure 9. NPR SWAA Supplementary information. (a) Visit count and **(b)** percent of attractive visits per worm of *npr* receptor mutants and *npr-10* rescue. **(c)** Transformed avoidance of *npr* mutants and *npr-10* rescue. **(d)** Amphid localization of *npr-10*::GFP in the head of a hermaphrodite. **(e)** Localization of *npr-10*::GFP in the tail of the hermaphrodite, with expression observed in the dorsal-rectal ganglia neurons: DVA, DVB, DVC, and ALN. Light grey denotes spatial controls ("S") (when applicable), dark grey denotes vehicle controls ("V"), colors denote ascr#8 values ("A") (*him-8*, purple; *e*, teal; *npr* mutants, blue; *npr-10*, sky blue; rescue, light blue). Error bars denote SEM**.** $n = 5$. * $p < 0.05$, ** $p < 0.01$, *** $p < 0.001$, **** $p < 0.0001$. **(a)** RM-ANOVA, followed by Bonferroni's Correction. **(b)** Paired *t*-test of V vs. A. **(c)** One-Way ANOVA, followed by Dunnett's Correction. ♂ denotes male data, ⚥ denotes hermaphrodite data.

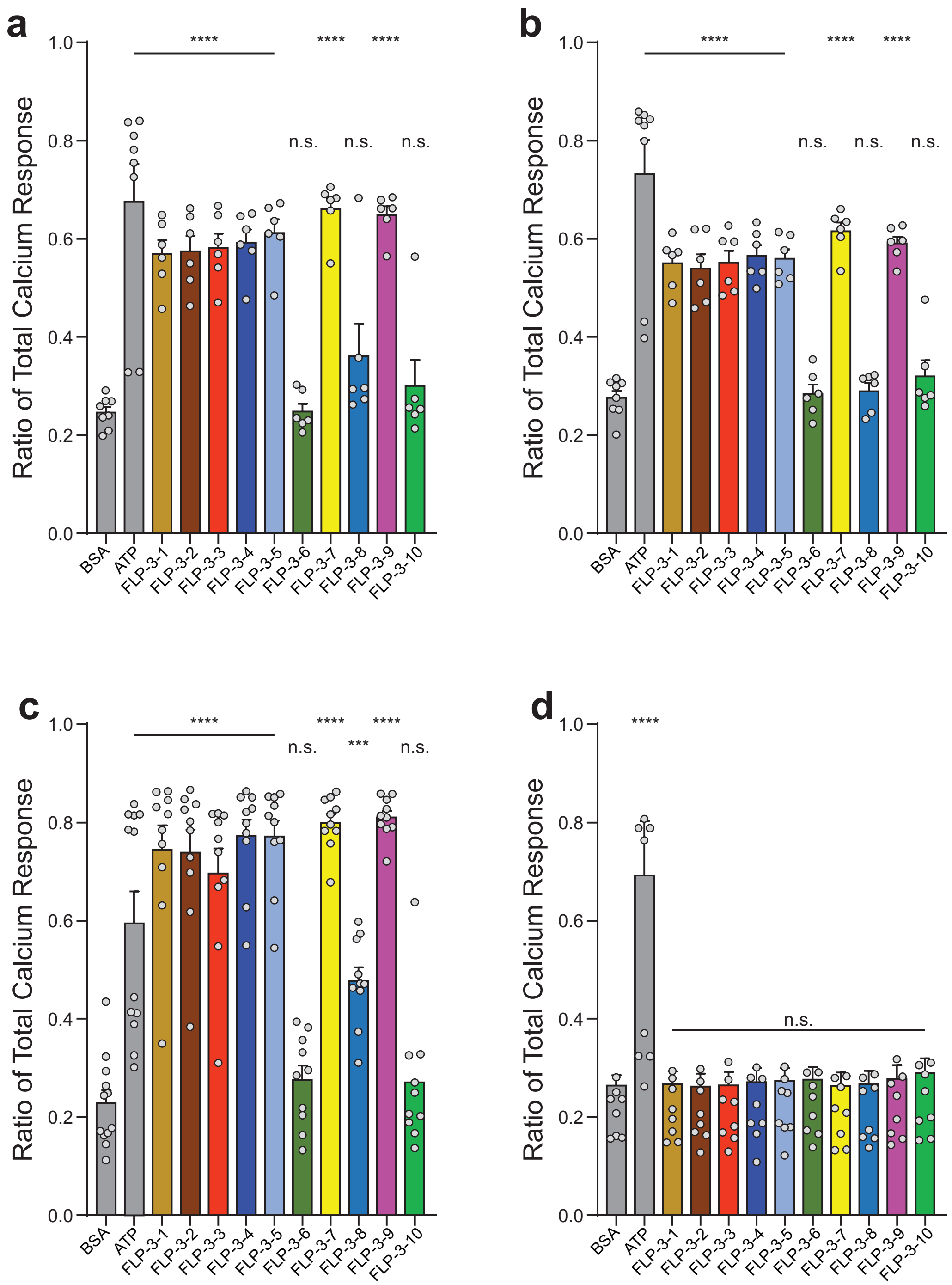

Supplementary Figure 10. FLP-3 Peptides Activate NPR-10 and FRPR-16 *in vitro*. Ratios of Total Calcium Response for cells transfected with **(a)** NPR-10A, **(b)** NPR-10B, **(c)** FRPR-16, and **(d)** empty vector control. Peptides FLP-3-6 and FLP-3-10 activated no receptors, likely due to lack of sequence homology. No peptides activated cells transfected with empty vector control. $n \geq 6$. **** $p < 0.0001$. One-Way ANOVA followed by Dunnett's Correction.

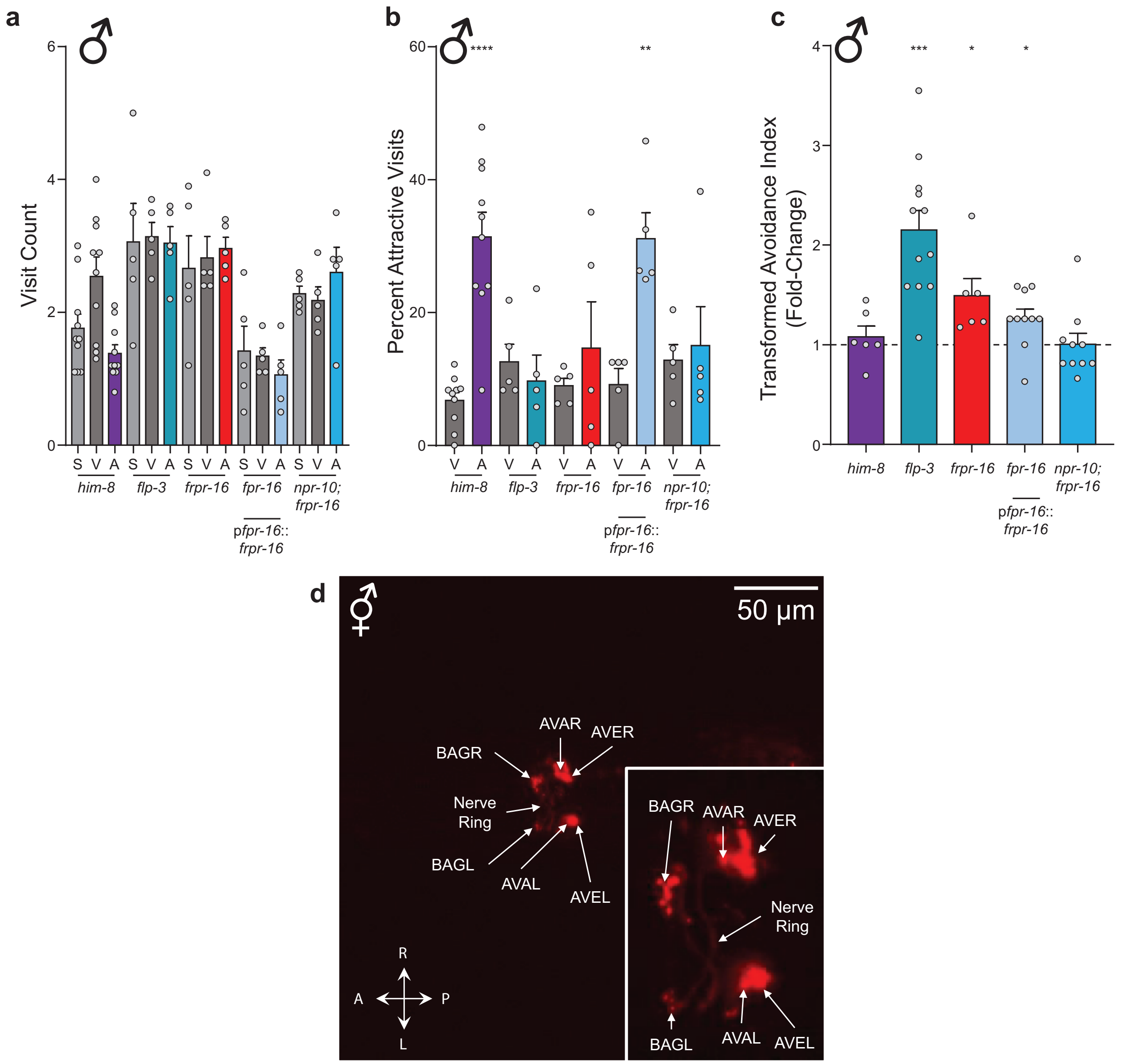

Supplementary Figure 11. FRPR-16 males display reduced behavioral response to ascr#8. (a) Visit count and **(b)** percent of attractive visits per worm of *frpr-16 lof* animals, transgenic rescues, and *frpr-16;npr-10* double mutant animals in a single worm attraction assay (SWAA). **(c)** Transformed avoidance values for *frpr-16 lof* animals, transgenic rescues, and *frpr-16;npr-10* double mutant animals. **(d)** Amphid localization of p*frpr-16*::*frpr-16*::SL2::mCherry in the head of a hermaphrodite. Similar expression within the AVA, AVE, AVD, and BAG neurons is seen. **(d, inset)** 2X zoom of main image, focusing on nerve ring localization. Light grey denotes spatial controls ("S") (when applicable), dark grey denotes vehicle controls ("V"), colors denote ascr#8 values ("A") (*him-8*, purple; *flp-3*, teal; *frpr-16*, red; rescue, light blue; *frpr-16;npr-10*, sky blue). Error bars denote SEM. $n = 5$. * $p < 0.05$, ** $p < 0.01$, *** $p < 0.001$, **** $p < 0.0001$. **(a)** RM-ANOVA, followed by Bonferroni's Correction. **(b)** Paired t-test of V vs. A. **(c)** One-Way ANOVA, followed by Dunnett's Correction. ♂ denotes male data, ⚥ denotes hermaphrodite data.

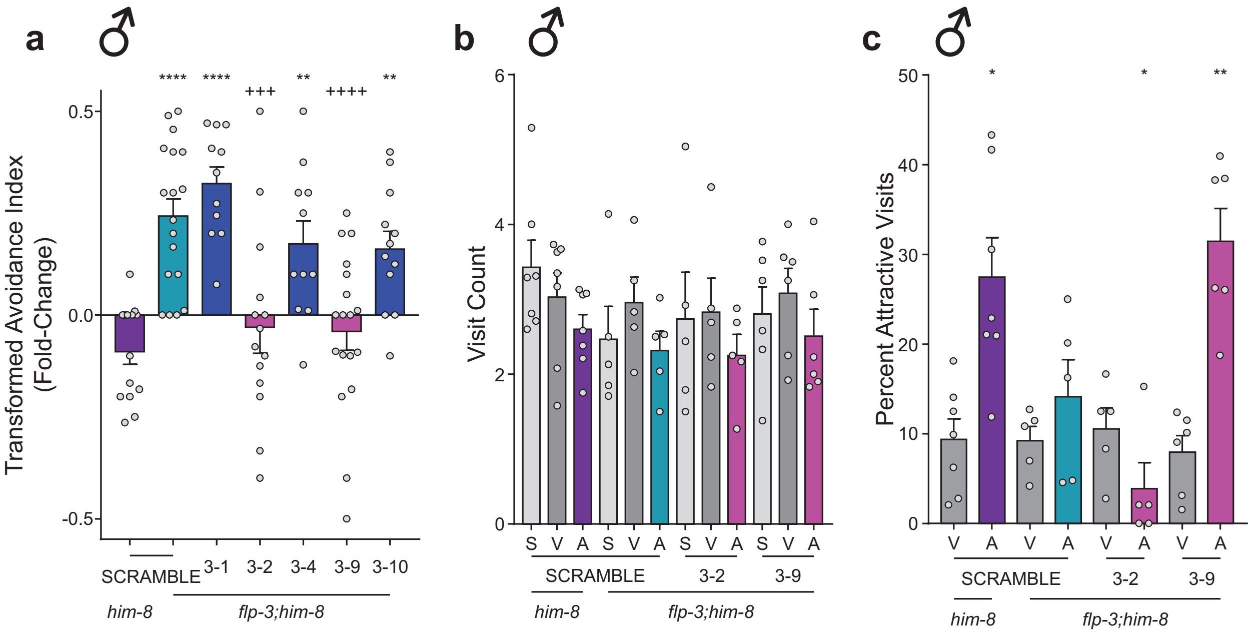

Supplementary Figure 12. Individual FLP-3 peptides rescue attraction to ascr#8. (a) Transformed avoidance of *flp-3* animals fed SCRAMBLE and different FLP-3 peptides. **(b)** Visit counts, and **(c)** percent of attractive visits per worm of *flp-3* animals fed peptides FLP-3-2, FLP-3-9. Light grey denotes spatial controls ("S") (when applicable), dark grey denotes vehicle controls ("V"), colors denote ascr#8 values ("A") (*him-8*, purple; *flp-3*, teal; FLP-3 peptides, pink). Error bars denote SEM**.** $n \geq 5$. * $p < 0.05$, ** $p < 0.01$, ***/+++ $p < 0.001$, ****/++++ $p < 0.0001$. **(a,b)** RM-Way ANOVA, followed by Bonferroni's Correction. **(c)** Paired *t*-test of V vs. A. ♂ denotes male data.

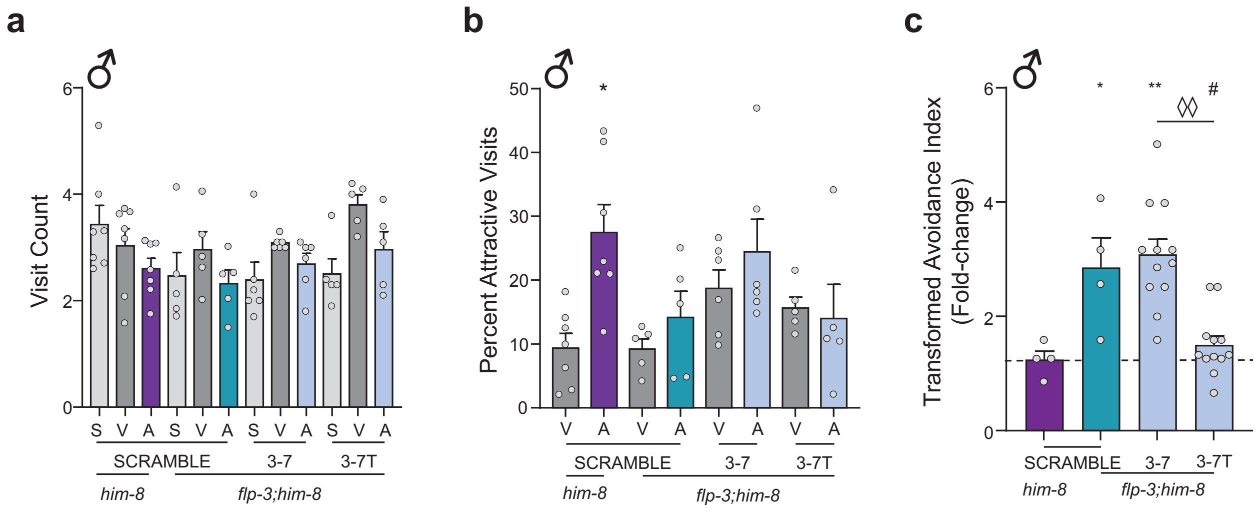

Supplementary Figure 13. Site-Directed Mutagenesis of FLP-3-7 confirms importance of the amino acid Threonine in Biological Activity. (a) Visit counts, and **(b)** percent of attractive visits per worm of *flp-3* animals fed FLP-3-7 peptides**. (c)** Normalized avoidance index of *flp-3* animals fed FLP-3-7 and FLP-3-7T confirms that the threonine is required and sufficient for the suppression of the ascr#8 avoidance response. Light grey denotes spatial controls ("S") (when applicable), dark grey denotes vehicle controls ("V"), colors denote ascr#8 values ("A") (*him-8*, purple; *flp-3*, teal; FLP-3-7 peptides, light blue). Error bars denote SEM**.** $n \geq 5$. * $p < 0.05$, **/◊◊ $p < 0.01$, *** $p < 0.001$, **** $p < 0.0001$. **(a)** RM-ANOVA, followed by Bonferroni's Correction. **(b)** Paired *t*-test of V vs. A. **(c)** One-Way ANOVA, followed by Dunnett's Correction. ♂ denotes male data.

Supplemental Table 1. Strains.

Strain	Genotype	Figure	Received From
N2	wild-type, Bristol	Fig. 1, Supp. Fig. 1, 2	*Caenorhabditis* Genetics Center (CGC)
CB4088	*him-5*(e1490)	Fig. 1, Supp. Fig. 1, 5, 7	CGC
CB1489	*him-8*(e1489*)*	Fig. 1, 2, 3, 5, 6, Supp Fig. 1, 2, 3, 4, 5, 7, 8, 9, 11, 12, 13	CGC
PT690	*osm-3*(mn391);*him-5*(e1490)	Fig. 1, Supp. Fig. 1, 2	Paul Sternberg (CalTech)
pk361	*flp-3*(pk361)	n/a	Chris Li (CUNY)
NY183	*flp-6*(pk1593)	n/a	Chris Li (CUNY)
NY106	*flp-12*(n4902)	n/a	Chris Li (CUNY)
NY193	*flp-19*(pk1594)	n/a	Chris Li (CUNY)
JSR99	*flp-3*(pk361);*him-8*(e1489)	Fig. 2, 3, 5, 6, Supp Fig. 3, 4, 5, 7, 9, 11, 12, 13	This Study
JSR1	*flp-6*(pk1593);*him-8*(e1489)	Fig. 2, Supp. Fig. 3	This Study
JSR4	*flp-12*(n4902);*him-8*(e1489)	Fig. 2, Supp. Fig. 3	This Study
JSR2	*flp-19*(pk1594);*him-8*(e1489)	Fig. 2, Supp. Fig. 3	This Study
VC2497	*flp-3*(ok3265)	n/a	CGC
JSR84	*flp-3*(ok3265); *him-8*(e1489)	Supp. Fig. 4	This Study
JSR109	*him-8*(e1489);worEx17[p*flp-3*::*flp-3*::GFP; p*unc-122*::RFP];*flp-3*(pk361)	Fig. 2, Supp. Fig. 3	This Study
JSR81	*him-8*(e1489);worEx17[pflp-3::flp-3::SL2::mCherry (25 ng/uL); punc-122::GFP (50 ng/uL)]	Fig. 2, Supp. Fig. 3	This Study
PS2218	*dpy-20*(e1362);*him-5*(e1490);syls33[HS.C3(50ng/uL) + pMH86(11ng/uL)]	n/a	Paul Sternberg (CalTech)

JSR113	*dpy-20*(e1362);*him-5*(e1490);syls33[HS.C3(50ng/uL) + pMH86(11ng/uL)];worEx27[p*flp-3*::*flp-3*::SL2::mCherry (25 ng/uL); p*unc-122*::GFP (50 ng/uL)]	Fig. 2	This Study
tm1782	*npr-4*(tm1782))	n/a	National BioResource Project (NBRP)
CX14393	*npr-5*(ok1583)	n/a	CGC
tm8982	*npr-10*(tm8982)	n/a	NBRP
VC4220	*frpr-16*(gk5305[loxP + p*myo-2*::GFP::*unc-54* 3' UTR + p*rps-27*::neoR::*unc-54* 3' UTR + loxP])	n/a	This Study
JSR91	*npr-4*(tm1782);*him-8*(e1489)	Fig. 3, Supp. Fig. 9	This Study
JSR97	*npr-5*(ok1583);*him-8*(e1849)	Fig. 3, Supp. Fig. 9	This Study
JSR102	*npr-10*(tm8982);*him-8*(e1489)	Fig. 3, Supp. Fig. 9	This Study
JSR103	*frpr-16*(gk5305[loxP + p*myo-2*::GFP::*unc-54* 3' UTR + p*rps-27*::neoR::*unc-54* 3' UTR + loxP]);*him-8*(e1489)	Fig. 5, Supp. Fig. 11	Vancouver Node of International *C. elegans* Knockout Consortium
JSR107	*npr-10(tm8982);frpr-16*(gk5305[loxP + p*myo-2*::GFP::*unc-54* 3' UTR + p*rps-27*::neoR::*unc-54* 3' UTR + loxP]);*him-8*(e1489)	Fig. 5, Supp. Fig. 11	This Study
JSR126	*npr-10*(tm8982);*him-8*(e1489);worEx37[p*npr-10*::*npr-10*::GFP (25 ng/uL); p*unc-122*::RFP (50 ng/uL)]	Fig. 3, Supp. Fig. 9	This Study

JSR133	*frpr-16*(gk5305[loxP + p*myo-2*::GFP::unc-54 3' UTR + p*rps-27*::neoR::*unc-54* 3' UTR + loxP]);*him-8*(e1489);worEx41[p*frpr-16*::frpr-16::SL2::mCherry (25 ng/uL); p*unc-122*::GFP (50 ng/uL)]	Fig. 5, Supp. Fig. 11	This Study
CU5248	*ceh-30*(tm272);*him-5*(e1490);smIs26[p*pkd-2*::GFP]	Supp. Fig. 7	Ding Xue (UC Boulder)
JSR98	*flp-3*(pk361);*ceh-30*(tm272);*him-5*(e1490);smIs26[p*pkd-2*::GFP]	Supp. Fig. 7	This Study
PT315	*egl-3*(n150);*him-8*(e1489)	Supp. Fig. 8	Maureen Barr (Rutgers)
PT440	*aex-5*(sa23);*him-8*(e1489)	Supp. Fig. 8	Maureen Barr (Rutgers)
PT436	*bli-4*(e937);*him-8*(e1489)	Supp. Fig. 8	Maureen Barr (Rutgers)

Supplemental Table 2. Plasmids

Plasmid ID	Function	Component	Array ID	Received From
pDONR p1-p2	DONR Vector	L1-L2 sites		
pL4440		Scramble Sequence		Victor Ambors, UMass Medical School
JSR#DKR20	ENTRY Clone	6xHis-MRFGKR-SCRAMBLE_KRK_STOP		
JSR#DKR27	ENTRY Clone	6xHis-MRFGKR-FLP3.1-KRK-STOP		
JSR#DKR22	ENTRY Clone	6xHis-MRFGKR-FLP3.2-KRK-STOP		
JSR#DKR12	ENTRY Clone	6xHis-MRFGKR-FLP3.4-KRK-STOP		
JSR#DKR13	ENTRY Clone	6xHis-MRFGKR-FLP3.9-KRK-STOP		
JSR#DKR23	ENTRY Clone	6xHis-MRFGKR-FLP3.10-KRK-STOP		
JSR#ANR39	ENTRY Clone	6xHis-MRFGKR-FLP-3-7-KRK-STOP		
JSR#ANR40	ENTRY Clone	6xHis-MRFGKR-FLP-3-7T-KRK-STOP		
pDEST-527	DEST Vector	*E. coli* Expression Vector		Dominic Esposito (Addgene plasmid # 11518)
JSR#DKR21	*E. coli* Expression	6xHis-MRFGKR-SCRAMBLE_KRK_STOP		
JSR#DKR28	*E. coli* Expression	6xHis-MRFGKR-FLP3.1-KRK-STOP		
JSR#DKR25	*E. coli* Expression	6xHis-MRFGKR-FLP3.2-KRK-STOP		
JSR#DKR15	*E. coli* Expression	6xHis-MRFGKR-FLP3.4-KRK-STOP		
JSR#DKR16	*E. coli* Expression	6xHis-MRFGKR-FLP3.9-KRK-STOP		

JSR#DKR26	*E. coli* Expression	6xHis-MRFGKR-FLP3.10-KRK-STOP		
JSR#ANR41	*E. coli* Expression	6xHis-MRFGKR-FLP-3-7-KRK-STOP		
JSR#ANR42	*E. coli* Expression	6xHis-MRFGKR-FLP-3-7T-KRK-STOP		
JSR#DKR18	Translational Fusion	p*flp-3*::*flp-3*::GFP	worE x17	
JSR#DKR34	Translational Fusion	p*flp-3*::*flp-3*::SL2::mCherry	worE x27	GeneWiz
JSR#DKR35	Translational Fusion	p*frpr-16*::*frpr-16*::SL2::mCherry	worE x41	
-	Co-Injection Marker	p*unc-122*::GFP		Mark Alkema, UMass Medical School
-	Co-Injection Marker	p*unc-122*::RFP		Shreekanth Chalasani, Salk Institute
DACR1432	DONR Vector	L2-L3 SL2::dsRed::*unc-54*3' UTR		Josh Hawk, Yale University
pPD95.75	GFP FireVector	GFP		

Supplemental Table 3. Primers

Primer/ Ultramer Name	Primer/Ultramer Sequence	References	Final Strain
p*flp-3* Forward	GACTCTGCAGcatttccaagacacatttgacg		JSR81, JSR95
flp-3 Reverse	GACTGGATCCttttccaaagcgcatggtt		JSR81, JSR95
p*npr-10* Forward	gtttgttttccggcactttc		
npr-10 Reverse_overhang	AGTCGACCTGCAGGCATGCAAGCTaatt gaacttggaatcgtggtagt		
p*npr-10* Forward#2	cggcactttcctcatttttc		JSR126, JSR137
p*frpr-16* Forward	accgatttctgatcgacgtg		
frpr-16 Reverse_overhang	CAGCAGTTTCCCTGAATTAAAATTAaac aattgccggagcttttc		
pfrpr-16 Forward#2	ttctgatcgacgtgttggtt		JSR133
GFP_C	AGCTTGCATGCCTGCAGGTCGACT	Boulin et al.	
GFP_D	AAGGGCCCGTACGGCCGACTAGTAG	Boulin et al.	
GFP/dsRed_D#2	GGAAACAGTTATGTTTGGTATATTGG G	Boulin et al.	
dsRed_E	TAATTTTAATTCAGGGAAACTGCTG		
dsRed_F	AAAGTTGgaaacagttatgtttgg		
SCRAMBLE Forward	GGGGACAAGTTTGTACAAAAAAGCAG GCTGGATGCGCTTTGGAAAACGTaattcg aagctccaccgcggtggcggccgctctagaactagtggatcc accggttccatggctagccacgcgcgtggatcccccgggct gcaggAAACGTaaataaCACCCAGCTTTCT TGTACAAAGTGGTCCCC		
FLP-3-1 Forward	GGGGACAAGTTTGTACAAAAAAGCAG GCTGGATGCGCTTTGGAAAACGTtctcca ctgggaacaatgcgctttggcAAACGTaaataaCAC CCAGCTTTCTTGTACAAAGTGGTCCCC		
FLP-3-2 Forward	GGGGACAAGTTTGTACAAAAAAGCAG GCTGGATGCGCTTTGGAAAACGTactcca ttgggaactatgcgttttggaAAACGTaaataaCACC CAGCTTTCTTGTACAAAGTGGTCCCC		
FLP-3-4 Forward	GGGGACAAGTTTGTACAAAAAAGCAG GCTGGATGCGCTTTGGAAAACGTaaccct cttggaaccatgcgctttggaAAACGTaaataaCACC CAGCTTTCTTGTACAAAGTGGTCCCC		

FLP-3-9 Forward	GGGGACAAGTTTGTACAAAAAA GCAG GCTGGATGCGCTTTGGAAAACG Taatcct gagaacgacacaccattcggaacaatgagatttgga AAA CGTaaataaCACCCAGCTTTCTTGT ACAAAGTGGTCCCC		
FLP-3-10 Forward	GGGGACAAGTTTGTACAAAAAA GCAG GCTGGATGCGCTTTGGAAAACG Ttctact gttgattcttcggagcccgtcattcgtgatcagAA ACGTa aataaCACCCAGCTTTCTTGTACA AAGTG GTCCCC		
FLP-3-7 Forward	GGGGACAAGTTTGTACAAAAAA GCAGGCTGGATGCGCTTTGGAA AACGTagtgcagagccattcggtactatgcgttt tggaAAACGTAAATAACACCCAG CTTTCTTGTACAAAGTGGTCCCC		
FLP-3-7T Forward	GGGGACAAGTTTGTACAAAAAA GCAGGCTGGATGCGCTTTGGAA AACGTagtgcaactccattcggtactatgcgtttt ggaAAACGTAAATAACACCCAGC TTTCTTGTACAAAGTGGTCCCC		
frpr-16-1 SeqValR	ggtatctgtggtttatgtcggatag		JSR103, JSR133
frpr-16-2 SeqValR	gaacggcatacgttcaggata		JSR103, JSR133
frpr-16-2 WT F	aaactaccctgtgcgattagg		JSR103, JSR133
frpr-16-1 WT R	CTTCCATACGTCAAGCACTTTC		JSR103, JSR133
frpr-16-1 SeqVal F	actttcaggcgaacacatact		JSR103, JSR133
pMYO-2 SEC Insertion	ccctcaatgtctctacttgt		JSR103, JSR133
NeoR-SEC Insertion	TTCCTCGTGCTTTACGGTATCG		JSR103, JSR133
