# Supplementary material for: Distinct neuropeptide-receptor modules regulate a sex-specific behavioral response to a pheromone
Source: Commun Biol. 2021 Aug 31;4:1018. doi: 10.1038/s42003-021-02547-7 (PMC8408276; doi:10.1038/s42003-021-02547-7)
Supplement: Supplementary file 3 — Description of Supplementary Files [file 42003_2021_2547_MOESM3_ESM.pdf]

Description of Additional Supplementary Files

File name: Supplementary Data 1

Description: Source data for all the figures in the manuscript.
